# Effects of Astragalus membranaceus on systemic lupus erythematosus in a mouse model of pregnancy

**DOI:** 10.1002/iid3.624

**Published:** 2022-05-11

**Authors:** Hong‐Qing Chen, Na Zhang, Cai‐Xia Li, Hong‐Xia Zhang

**Affiliations:** ^1^ Department of Obstetrics Hengshui Fourth People's Hospital Hengshui Hebei China; ^2^ Department of Clinical Pharmacy The Fourth Hospital of Shijiazhuang Shijiazhuang Hebei China; ^3^ The Fourth Hospital of Shijiazhuang Shijiazhuang Hebei China; ^4^ Department of Pharmacy The Fourth Hospital of Shijiazhuang Shijiazhuang Hebei China

**Keywords:** astragalus membranaceus (AM), helper T cell 17 (Th17) differentiation, lupus nephritis (LN), systemic lupus erythematosus (SLE)

## Abstract

**Background:**

This study used astragalus membranaceus (AM) to treat systemic lupus erythematosus (SLE) model mice during pregnancy, aiming to explore the role of AM in Helper T cell 17 (Th17) differentiation and SLE during pregnancy.

**Methods:**

We used lipopolysaccharide to constructed the SLE mouse model. AM decoction given by intragastric administration lasted from the eighth week of the mouse age until the mouse was killed. We estimated the messenger RNA  levels of IL‐17a and Rorc, counted the Th17 cell number and examined the levels of cytokines including interleukin (IL)‐12, tumor necrosis factor α, interferon gamma, IL‐17A in mouse serum. Periodic acid‐Schiff staining and renal glomerular/tubulointerstitial (TI) score were used to evaluate the progression of lupus nephritis (LN).

**Results:**

AM treatment improved the conception rate and increased the number and average weight of fetuses in SLE mice. It significantly decreased the urinary albumin/creatinine ratios and reduced the glomerular scores and TI scores in the pregnant SLE mice. AM gavage significantly decreased the weight of spleen, mesenteric lymph node, total splenocytes and T cells, and the expression of proinflammatory factors. Furthermore, AM treatment reduced the ratio of Th17 cells and the expression levels of RORγt and IL‐17A.

**Conclusion:**

AM significantly improved pregnancy outcomes and inhibited lupus nephritis during pregnancy in SLE mice.

## Graphical abstract

This study used astragalus membranaceus (AM) to treat systemic lupus erythematosus model mice during pregnancy and found that AM significantly improved pregnancy outcomes and inhibited lupus nephritis and the secretion of inflammatory factors during pregnancy. The underlying mechanism may be achieved by inhibiting the differentiation of Th17 cells.

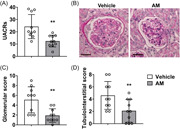

## 1 INTRODUCTION

Systemic lupus erythematosus (SLE) is a chronic autoimmune disease with multiple system damage, which occurs in women of childbearing age.^1^ With the improvement of the efficacy of SLE, more and more women with SLE choose pregnancy.^2^ SLE itself does not damage fertility, but lupus nephritis (LN) and the use of cytotoxic drugs may reduce fertility.^3^ Pregnancy with SLE is a high‐risk pregnancy.^4^ Pregnancy will increase the risk of recurrence of SLE.^5^ The disease itself will also increase the possibility of adverse pregnancy outcomes for pregnant women, mainly including pre‐eclampsia, hypertension of pregnancy, premature birth, fetal loss, fetal intrauterine growth restriction, neonatal lupus.^6^

Helper T cell 17 (Th17) is a CD4+ T cell subgroup characterized by the production of proinflammatory factor interleukin (IL)‐17.^7^ There is a large amount of literature showing that Th17 cells are involved in the pathogenesis of SLE and play an important role in inducing inflammation and organ damage.^8^ In SLE, the number of Th17 cells increases and the function is hyperactive.^9^ Th17 cells recruit inflammatory cells by secreting proinflammatory cytokines and expands the inflammatory responses.^10^ In addition, Th17 cells enhance the humoral immune responses and ultimately lead to target organ damage.^11^ It has been clinically found that traditional Chinese medicine has unique effects in the treatment of SLE with increased efficacy and attenuated toxicity and fetus during pregnancy.^12^ Therefore, increasing SLE patients choose traditional Chinese medicine for treating SLE during pregnancy.

As a classic Chinese medicine, astragalus membranaceus (AM) is widely recognized and has been comprehensive and in‐depth research.^13^ AM is the root of the perennial herb AM (Fisch.) Bge.var. Mongholicus (Bge.) Hsiao and AM (Fisch.) Bge.^14^ The main components of AM are saponins, polysaccharides, and flavonoids, which have a variety of biological functions such as immune regulation, antitumor, antivirus, antiaging, and hypoglycemic.^15^ In the treatment of SLE, AM could increase the curative effect, reduce the side effects of glucocorticoid and reduce the infection through multiple ways of immunomodulation.^16^ Studies have shown that AM could regulate the balance of Treg/Th17 cells.^17^ This study used AM to treat SLE model mice during pregnancy, aiming to explore the role of AM in Th17 cell differentiation and SLE during pregnancy.

## 2 METHODS

### 2.1 Reagents

AM was ordered from Furuibang Chinese Medicine Co., Ltd. AM decoction was prepared according to standard procedures.^18^ Briefly, 100 g AM roots were refluxed with water (1:8, wt/vol) for 1.5 h. The mixture was sonicated for 30 min and then extracted. The extract was concentrated under reduced pressure to 100 ml, so that the residual AM concentration was 1 g/ml. Lipopolysaccharide (LPS) was ordered from Sigma‐Aldrich.

### 2.2 Animals

MRL/lpr mice weighing between 20 and 25 g (aged 6 weeks) were housed in the facility with standard light/dark cycle and constant temperature and humidity. All mouse experiments conducted in this study were conducted in strict accordance with animal ethics guidelines. All institutional and national guidelines for the care and use of laboratory animals were followed and approved by the Fourth Hospital of Shijiazhuang. LPS was used for the construction of SLE mouse model according to standard protocol.^19^ Briefly, female MRL/lpr mice (aged 6 weeks) were intraperitoneally (i.p.) administered with LPS (30 μg/kg). Saline was used as the negative control.

### 2.3 Grouping and intervention

SLE mice were randomized into two groups with 40 mice per group. AM group and Vehicle group. The mice in AM group were provided with AM decoction by intragastric administration once daily. AM treatments lasted from the eighth week of the mouse age until the mouse was killed. Distilled water was used as a negative control. The mice became pregnant at the tenth week of age. After the quantification of conception, 10 pregnant mice were randomly selected from each group for further study, and the remaining pregnant mice were euthanized and not analyzed in the further study. At the time of pregnancy E18.5, the mice were euthanized and the mesenteric lymph node (MLN) of the spleen, kidney, and MLNs were collected for analysis.

### 2.4 Quantitative real‐time PCR

Total RNA was extracted by RNeasy Mini Kit (74104, Qiagen) and transcribed to complementary DNA by FastKing RT Kit (With gDNase) (TIANGEN, KR116). QRT‐PCR analysis was performed by FastKing One Step RT‐qPCR Kit (SYBR Green) purchased from (TIANGEN, FP313). The 2^−∆∆^CT method was used to calculate the relative expression of the target gene to be tested. Ppib was used as a negative control. The level of *Ppib* was used as internal control. The primers used in this study are as follows:

*IL17a*F: CAGGGAGAGCTTCATCTGTGT

*IL17a*R: GCTGAGCTTTGAGGGATGAT

*Rorc*F: CCGCTGAGAGGGCTTCAC

*Rorc*R: TGCAGGAGTAGGCCACATTACA

*Ppib*F: TGGAGAGCACCAAGACAGACA

*Ppib*R: TGCCGGAGTCGACAATGAT

### 2.5 Fluorescence activated cell sorting

The spleen of mice and the MLN (submaxillary, thoracic, axillary, renal, and mesenteric) in spleen were weighted at E18.5. The removed mouse spleen was washed with fluorescence‐activated cell sorting buffer and digested into single cells. The total spleen cell number was calculated by counting a portion of the cells diluted by flow cytometry. Pharm Lyse lysis buffer was used for the lysis of red cells in spleen. Cells were counted and adjusted to a concentration of 1 × 10^7^/ml by Flow Cytometry Staining Buffer. Surface antibodies (CD4‐APC, RoRγt‐PE, IL‐17A‐Amcyan) were added to the cell suspension. The solution was incubated for 15 min at room temperature in the dark. Flow staining buffer (500 μl) was used to wash and resuspend the cells for the testing on the machine.

### 2.6 Albumin/creatinine examination

The urinary albumin was determined by Mouse Albumin ELISA Kit (ab207620; Abcam). The creatinine in mouse blood and urinary was evaluated by Creatinine Assay Kit (ab65340; Abcam) following the instruction. UACRs (urinary albumin/creatinine ratios) were used to represent the kidney function.

### 2.7 Periodic acid‐Schiff stain (PAS)

The mouse tissues fixed in paraffin were sliced by a cryostat. Periodic acid (HIO_4_·2H_2_O), Schiff stain (basic fuchsin 0.5 g, H_2_O 100 ml) and hematoxylin were used for PAS staining of tissue sections according to standard procedures.^19^ The ratio of the glomerular PAS‐positive area to the glomerular cluster area was defined as the mesangial matrix score.

### 2.8 Enzyme linked immunosorbent assay (ELISA)

The levels of cytokines including IL‐12, tumor necrosis factor α  (TNF‐α), interferon gamma (IFN‐γ), IL‐17A were examined by commercial ELISA kits: Mouse IL‐12 p70 ELISA Kit (ab119531; Abcam), Mouse TNF alpha ELISA Kit (ab208348; Abcam), Mouse IFN gamma ELISA Kit (ab282874; Abcam) and Mouse IL‐17A ELISA Kit (ab283537; Abcam) according to the instructions.

### 2.9 Statistical analysis

Data were presented as mean and standard deviation (mean ± *SD*). The significant difference was analyzed using two‐tailed *t* test in GraphPad prism 7. *p* < .05 were regarded as significant difference. It was considered statistically significant when *p* value was less than 0.05.

## 3 RESULTS

### 3.1 Treatment of AM alleviates pregnancy outcomes in pregnant SLE mice

**Figure 1:**
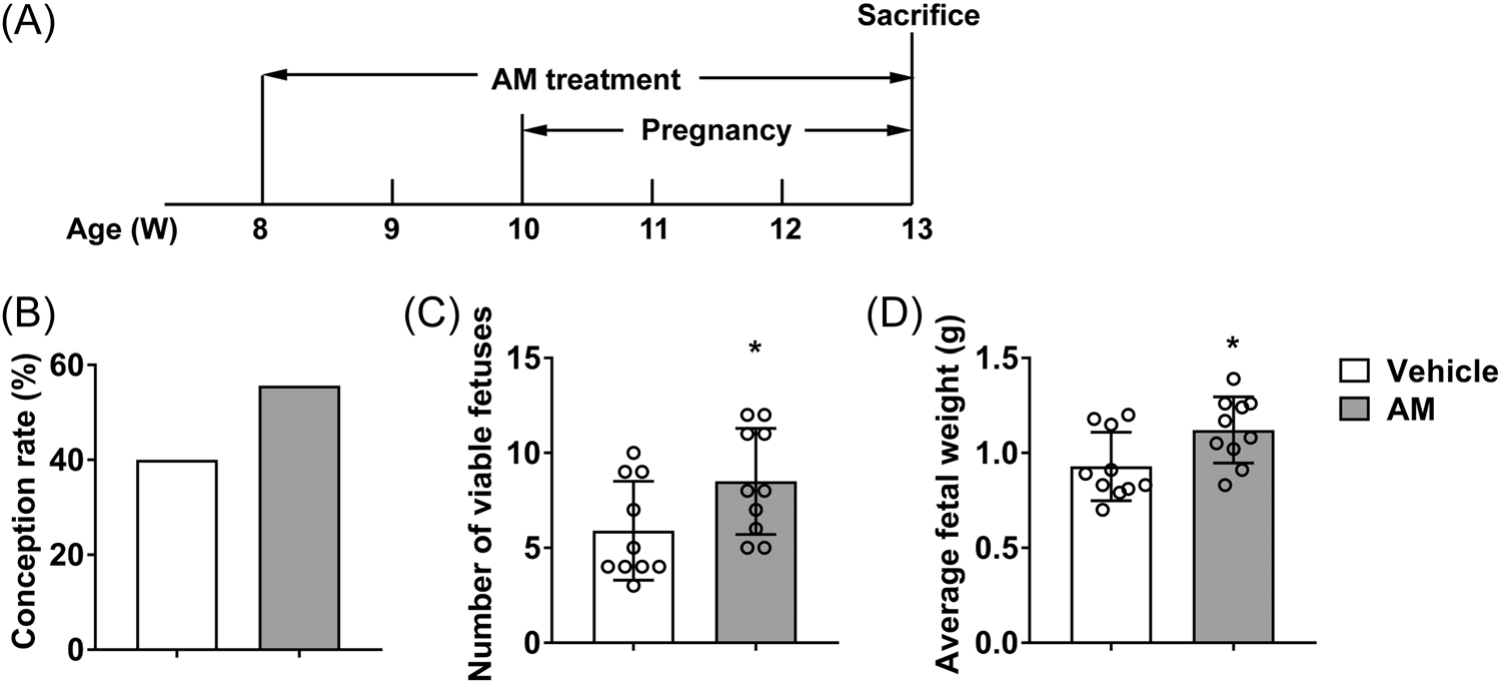
Pregnancy outcomes in SLE mice treated with AM. (A) The schematic diagram of animal model. (B) The conception rate of SLE mice, 40 mice per group. (C,D) The number (C) and average weight (D) of fetuses from each pregnant mouse. Ten pregnant mice per group. AM,astragalus membranaceus; SLE, systemic lupus erythematosus

To demonstrate the protective effects of AM on pregnant SLE mice, we used AM decoction to gavage SLE mice. The animal experiment process and drug treatment time are shown in Figure 1A. Eight‐week‐old SLE mice received AM or distilled water gavage treatment. The drug treatment continued until the animal was killed. SLE mice become pregnant at 10 weeks of age. The pregnant mice were killed at 18.5 days of gestation. AM gavage improved the conception rate of SLE mice (Figure 1B). The number (Figure 1C) and average weight (Figure 1D) of fetuses in the SLE mice of AM treated group increased significantly (*p* < .05) compared with the vehicle group. These figures demonstrated that AM treatment improved the pregnancy outcomes of SLE mice statistically.

### 3.2 Treatment of AM alleviates renal injury in pregnant SLE mice

**Figure 2:**
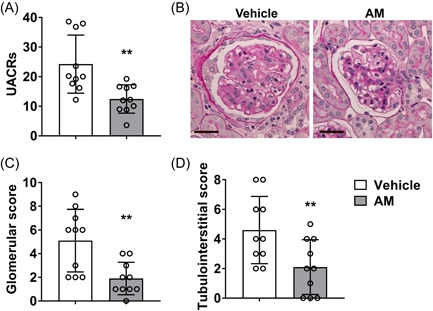
AM attenuated lupus nephritis (LN) in pregnant SLE mice. (A) The UACRs (urinary albumin/creatinine ratios) in pregnant MRL/lpr mice. (B) Representative images of glomerular periodic acid‐schiff (PAS) staining. The scale bar is 25 μm. (C) Renal glomerular score. (D) Renal tubulointerstitial score. 10 pregnant mice per group. AM,astragalus membranaceus; SLE, systemic lupus erythematosus

To evaluate the kidney function in pregnant SLE mice, we detected he urinary albumin and creatinine levels and slice, stain and score the kidneys. Our results revealed that AM treatment significantly decreased the UACRs in the pregnant MRL/lpr mice (Figure 2A). The pathological sections after Acid‐Schiff (PAS) staining were shown in Figure 2B. Our results showed that the glomeruli of SLE mice were infiltrated with inflammatory cells, the volume increased, the renal tubules were swollen, and casts were visible in the lumen. After AM treatment, the infiltration of glomeruli and renal interstitial inflammatory cells in the mice was significantly reduced compared with the SLE group. The renal tubules were slightly turbid and swollen, and no cast was seen. In addition, AM treatment significantly reduced the glomerular scores (Figure 2C) and tubulointerstitial scores (Figure 2D) compared to SLE mice. Taken together, our data suggest that AM relieves the renal injury in MRL/lpr mice.

### 3.3 The effect of AM on inflammation in pregnant SLE mice

**Figure 3:**
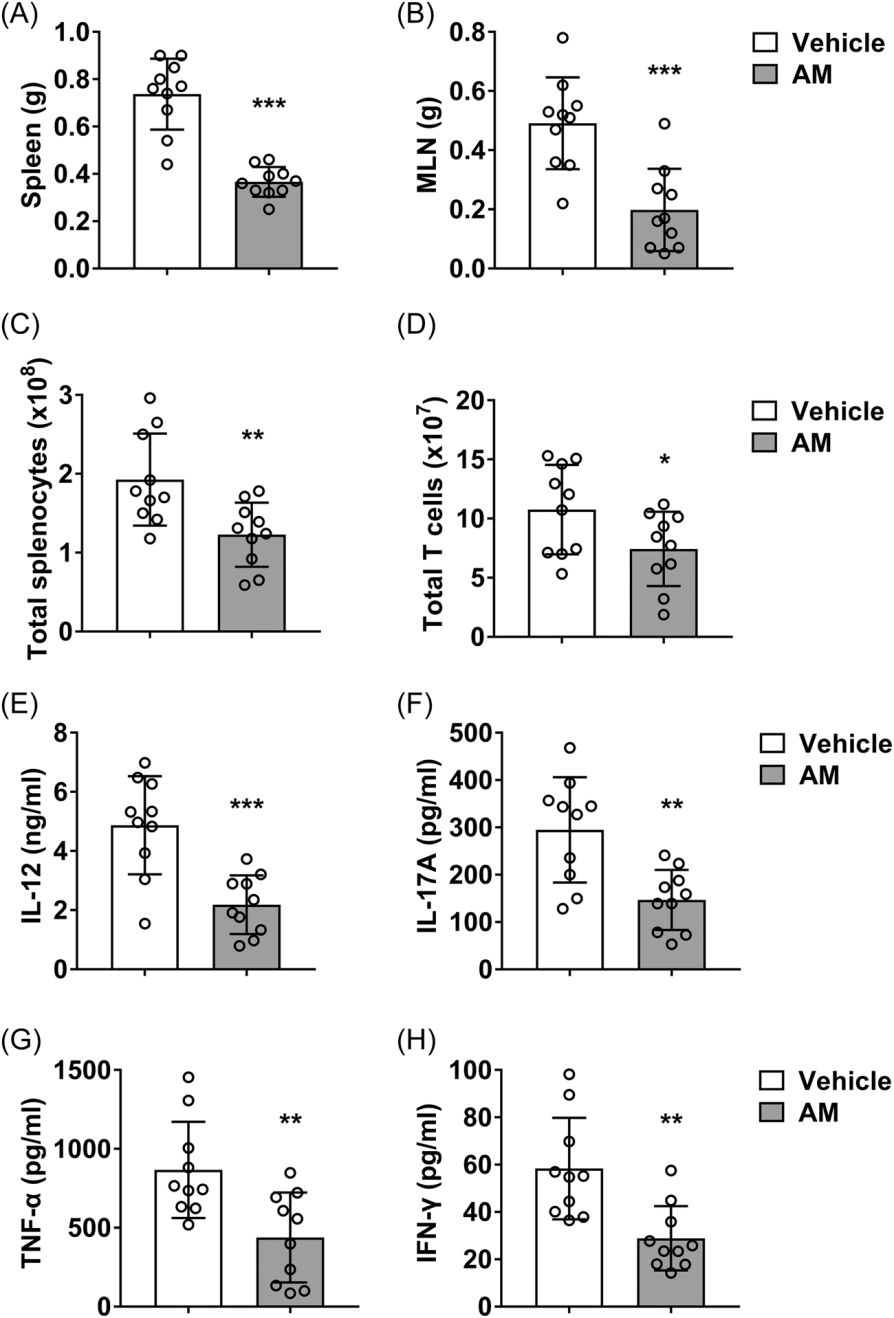
The effect of AM on inflammation in pregnant SLE mice. (A, B) The weight of spleen and MLN. (C,D) Total cells number (C) and T cells number (D) in spleen. (E–H) Serum IL‐12 (E), IL‐17A (F), TNF‐α (G), and IFNγ (H) protein level. 10 pregnant mice per group. AM, astragalus membranaceus; IFNγ, interferon gamma; IL, interleukin; SLE, systemic lupus erythematosus

To further investigate the effect of AM on inflammation in pregnant SLE mice, we obtained the spleens of SLE mice immediately after euthanizing and performed relevant analysis. We demonstrated that AM gavage significantly decreased the weight of spleen (Figure 3A) and MLN (Figure 3B) of SLE mice (*p* < .001), suggesting that AM treatment inhibited the immune activation induced by LPS. Moreover, AM administration significantly inhibited the upregulation of total splenocytes (Figure 3C, *p* < .01) and T cells (Figure 3D, *p* < .05) induced by SLE. The serum IL‐12 (Figure 3E, *p* < .001), IL‐17A (Figure 3F, *p* < .01), TNF‐α (Figure 3G, *p* < .01), and IFNγ (Figure 3H, *p* < .01) protein levels all decreased statistically post AM treatment compared with the vehicle group. These figures suggested that AM gavage significantly inhibited the abnormal activation of immunity of SLE mice.

### 3.4 AM inhibited the differentiation of Th17 cells in vivo

**Figure 4:**
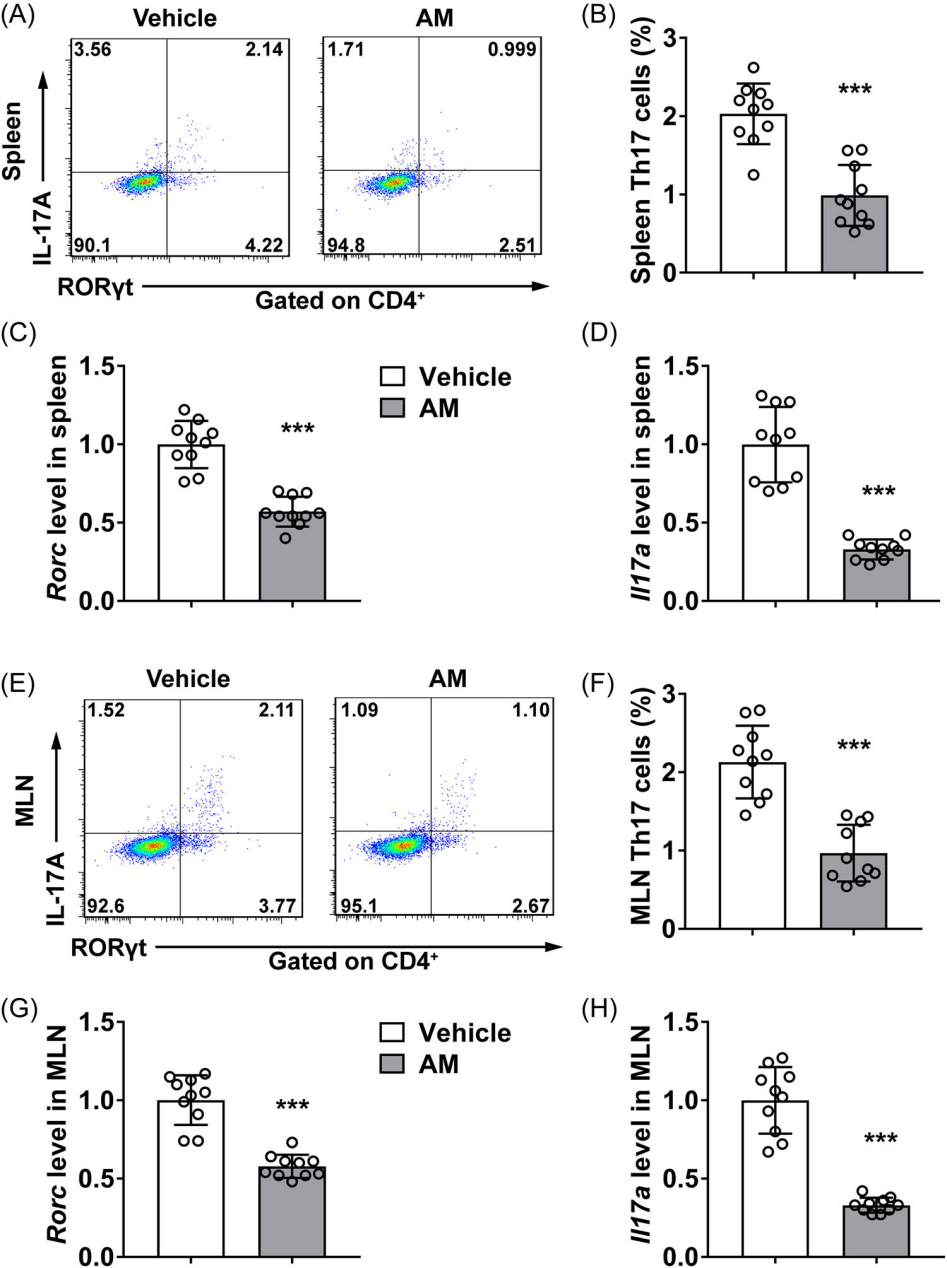
AM inhibited the differentiation of Th17 cells in vivo. (A,B) Analysis of Th17 cells population in spleen by FACS. (C,D) The mRNA level of *Rorc* (C) and *IL‐17A* (D) in spleen. (E,F) Analysis of Th17 cells population in MLN by FACS. (G,H) The mRNA level of *Rorc* (G) and *IL‐17A* (H) in MLN. 10 pregnant mice per group. AM, astragalus membranaceus; IL, interleukin; mRNA, messenger RNA

Th17 cells play an important role in the progression of SLE. To demonstrate that AM administration can regulate Th17 cell levels in SLE mice, we performed flow cytometric analysis. As presented in Figure 4A,B, AM gavage statistically inhibited the upregulation of Th17 cells in the spleen of mice post LPS treatment (*p* < .001). Consistently, the Rorc (coding gene of RORγt, Figure 4C) and IL‐17a (Figure 4D) messenger RNA (mRNA) levels in the spleen of SLE mice both decreased dramatically post AM administration (*p* < .001). Similarly, AM gavage also induced the downregulation of Th17 cells in the MLN of SLE mice (Figure 4E,F, *p* < .001). The Rorc (Figure 4G) and IL‐17a (Figure 4H) mRNA levels in the MLN of SLE mice also decreased statistically in AM group compared to vehicle group (*p* < .001). These results indicated that AM significantly inhibited Th17 differentiation in SLE mice.

## 4 DISCUSSION

In recent years, the chemical composition and pharmacological activities of AM have been studied extensively. Pharmacological experiments and clinical reports have shown that Astragalus has an important role in immune regulation, antitumor, antivirus, diabetes, and antiaging.^13^ Component analysis shows that AM contains a variety of active ingredients, including astragalus polysaccharides, astragalus saponins, and astragalus flavonoids.^20^ As a class of biological macromolecular components, Astragalus polysaccharides have extensive research on its structure and activity. It has immunomodulatory, antitumor, antiatherosclerosis, hypoglycemic, antiviral, and antiaging activities, and could treat metabolic disorders and delayed neurodegenerative diseases. Studies have shown that Astragalus polysaccharides could improve the symptoms of early diabetic nephropathy by affecting the expression of  nuclear factor kappa B and IκB in the renal cortex.^21^ Astragalus saponins have a variety of pharmacological activities, mainly including immune regulation, antitumor, hypoglycemic, antivirus, and multiple organ protection. In the study of the protection of multiple organs, it was found that the total glycosides of astragalus could protect the kidney from oxidative damage induced by shock waves.^22^ The total flavonoids of Astragalus are another active part of Astragalus, which has the activities of immune regulation, anti‐injury, anti‐mutation, antitumor, and inhibiting atherosclerosis. For SLE, the study found that AM could affect the activity of natural killer cells of peripheral blood mononuclear cells in patients with SLE.^23^ Large‐dose AM injection could reduce the infection rate and urine protein of patients with LN, and improve immune function.^16^ In this article, we found that AM relieves SLE by repressing Th17 cells differentiation in pregnant MRL/lpr mice. However, it is not clear which component of Astragalus is the key component to alleviate SLE, and this effect may be a composite effect of multiple factors, rather than a single compound.

SLE is a complex autoimmune disease that impaired multiple organs and systems. It seriously affects the quality of life of humans. SLE presents a variety of immune abnormalities. The main clinical feature of SLE is the immune complex deposition and inflammation/necrosis in multiple tissues and organs such as kidneys, skin, blood vessels, and central nervous system.^24^ The pathogenesis of SLE is a disorder of the patient's immune balance caused by the combined action of genetic factors and environmental factors. A large number of studies have shown that autoantibodies produced by disordered B cells, abnormal expression and function of T cells, and cytokines secreted by abnormal T cells are all involved in the pathogenesis of SLE.^25^ Th17 cells are an effector CD4+ T cell subset characterized by the secretion of IL IL‐17A.^26^ It has different functional characteristics from the traditional T cell subsets Th1 and Th2 cells. Th17 cells are a proinflammatory subgroup of Th cells and play a crucial role in the pathogenesis of autoimmune diseases. The powerful proinflammatory activity of Th17 cells may lead to many pathological features of SLE, such as induction of vascular inflammation, leukocyte recruitment, B cell activation, and the production of autoantibodies.^27^ A large number of studies have shown that the number of Th17 cells and IL‐17A level in the peripheral blood of SLE patients have increased, and the upregulation of Th17 cells has also been found in the skin lesions, lungs, and kidneys of these patients.^28^ It has been reported that Th17 cells may be involved in the abnormal immune function of autoimmune diseases such as SLE in recent years, but the specific mechanism of their role in SLE is still not fully understood.^29^ Accumulating evidence has demonstrated that Th17 cells may be involved in nephritis, vasculitis, central nervous system infection and the production of autoantibodies in SLE patients.^30^ Therefore, researches on Th17 cells and related cytokines have shown that Th17 has great application prospects for targeted therapy of SLE. In this article, we found that AM reduced the T cell number in the SLE model mouse spleen and the levels of IL‐12, IL‐17A, TNF‐α, and IFNγ in serum. In addition, we found that after AM treatment, the ratio of Th17 cells (CD4+RORγt+IL17A+), and the expression levels of RORγt and IL‐17A were significantly reduced, indicating that AM significantly inhibited the differentiation of Th17 cells.

IL‐17A is a characteristic factor secreted by Th17 cells. IL‐17A can induce the secretion of interleukins (IL‐1β, IL‐6), tumor necrosis factor α (TNF‐α), colony stimulating factors (G‐CSF, GM‐CSF) and other inflammatory cytokines.^31^ These factors can recruit a variety of effector cells including neutrophils and B cells to release various cytokines and a large number of autoantibodies, and ultimately lead to end‐organs and tissues dominated by nephritis and vasculitis damage.^31^ Animal experiments have shown that the knockout of IL‐17A gene in lupus mice leads to a decrease in the number of peripheral blood B cells and serum total immunoglobulin G (IgG) levels, suggesting that IL‐17A participates in the pathogenesis of SLE by enhancing the humoral immune response.^32^, ^33^ A number of independent studies have proved that the serum IL‐17A level and the number of Th17 cells in new‐onset SLE patients are higher than healthy people regardless of the type of SLE and the age of its onset.^34^ Studies have also shown that although the serum IL‐17A level of SLE patients is higher than that of healthy people, it is not significantly related to disease activity. The decrease in IL‐17A expression after treatment suggests that the controversy may be related to medicine application. In specific skin rashes of discoid lupus erythematosus, subacute cutaneous lupus erythematosus and SLE patients, it was found that T cells infiltrated in the skin tissues highly express IL‐17A.^35^ Compared with skin type and SLE, Th17 hyperfunction is more closely related to LN. The level of IL‐17A in peripheral blood and urine sediment of LN patients is not only higher than that of healthy people, but also higher than that of non‐LN SLE patients.^36^ At the same time, this high expression is more likely to occur in patients with severe renal biopsy pathology. IL‐17A can mediate early renal functional and histological damage through humoral immunity. There is an abnormal increase of IL‐17A in the kidney tissue of patients with LN, which is positively correlated with the renal histological activity index but negatively correlated with the chronic index.^37^ Summers et al.^33^ also found that after knocking out the IL‐17A gene, the accumulation of IgG, IgM and complement C3 in the kidney was reduced, and the proportion of abnormal glomeruli was significantly reduced. In this article, we found that AM attenuated LN in pregnant SLE mice, which may be achieved by inhibiting Th17 cells.

In conclusion, this study used AM to treat SLE model mice during pregnancy and found that AM significantly improved pregnancy outcomes and inhibited LN and the secretion of inflammatory factors during pregnancy. The underlying mechanism may be achieved by inhibiting the differentiation of Th17 cells.

## CONFLICTS OF INTEREST

The authors declare no conflicts of interest.

## ETHICS STATEMENT

All institutional and national guidelines for the care and use of laboratory animals were followed and approved by the Fourth Hospital of Shijiazhuang.

## Data Availability

Data could be obtained upon reasonable request to the corresponding author.
